# Guilt leads to enhanced facing-the-viewer bias

**DOI:** 10.1371/journal.pone.0195590

**Published:** 2018-04-12

**Authors:** Mowei Shen, Chengfeng Zhu, Huayu Liao, Haihang Zhang, Jifan Zhou, Zaifeng Gao

**Affiliations:** Department of Psychology, Zhejiang University, Hangzhou, China; School of Psychology, CHINA

## Abstract

As an important moral emotion, guilt plays a critical role in social interaction. It has been found that people tended to exhibit prosocial behavior under circumstances of guilt. However, all extant studies have predominantly focused on the influence of guilt on macro-level behavior. So far, no study has investigated whether guilt affects people’s micro-level perception. The current study closes this gap by examining whether guilt affects one’s inclination to perceive approaching motion. We achieved this aim by probing a facing-the-viewer bias (FTV bias). Specifically, when an ambiguous walking biological motion display is presented to participants via the point-light display technique, participants tend to perceive a walking agent approaching them. We hypothesized that guilt modulated FTV bias. To test this hypothesis, we adopted a two-person situation induction task to induce guilt, whereby participants were induced to feel that because of their poor task performance, their partner did not receive a satisfactory payment. We found that when participants were told that the perceived biological motion was motion-captured from their partner, the FTV bias was significantly increased for guilty participants relative to neutral participants. However, when participants were informed that the perceived biological motion was from a third neutral agent, the FTV bias was not modulated by guilt. These results suggest that guilt influences one’s inclination to perceive approaching motion, but this effect is constrained to the person towards whom guilt is directed.

## Introduction

As a type of moral emotion, guilt is triggered when one realizes that one’s behavior violates social moral standards and causes harm to other people [1,2]. However, although guilt is a negative emotional state [3] (for example, guilt elicits feelings of remorse and tension), it functions to promote prosocial behavior by avoiding transgressions and maintaining healthy social bonds between people. Guilty individuals have tendencies to reparative actions, such as confessions, apologies, and attempting to reduce harm, which are beneficial in restoring the relationship between the transgressor (person in a guilty state) and the victim. Therefore, guilt is ecologically and psychologically important to our social life.

So far, much attention has been paid to guilt-related prosocial behavior, focusing on the relationship between state or trait guilt and decision-making or external behavior. It has been found that guilty individuals are inclined to perform prosocial behavior to compensate the harm to their victims [3–5], even at the expense of benefits to a third person[3]. In the absence of an opportunity for compensation, guilt may lead to self-punishment as a sign of remorse [6,7]. Additionally, dispositional guilt is positively correlated with prosocial orientation [8,9]. On the other hand, Cavalera and Pepe [10] recently revealed certain drawbacks of guilt on cognitive processing, by showing that guilt impairs working memory performance compared to the neutral affective state.

However, no study so far has explored whether guilt affects visual perception, in sharp contrast to the status quo understanding, revealed by a plethora of studies, that affective states have a comprehensive impact on visual perception [11]. As a first step towards bridging this gap, here we explored whether a guilty state influenced perception of approach/avoidance.

According to the multi-component emotion model, the motivational component of emotion elicits adaptive action tendencies [12,13] (active approach *vs*. passive avoidance). It is suggested that the approach/avoidance motivational component underlies the link from emotion to behavior. Guilt-induced prosocial behavior, therefore, may be initiated by the approach/avoidance motivational system. This approach/avoidance motivational component may further exert a top-down influence on approach/avoidance perception. Specifically, while guilt leads one to focus on the harm caused by one’s improper behavior, it is suggested that a goal of compensating the victim, which ultimately leads to prosocial behavior, is also formed. This guilt-induced compensation goal may modulate perception of approach, since approach offers the guilty person more opportunity to restore the impaired relationship. Furthermore, considering that the feeling of guilt is constrained to the victim, the impact of guilt on perception of approach is potentially victim-specific.

**Fig 1:**
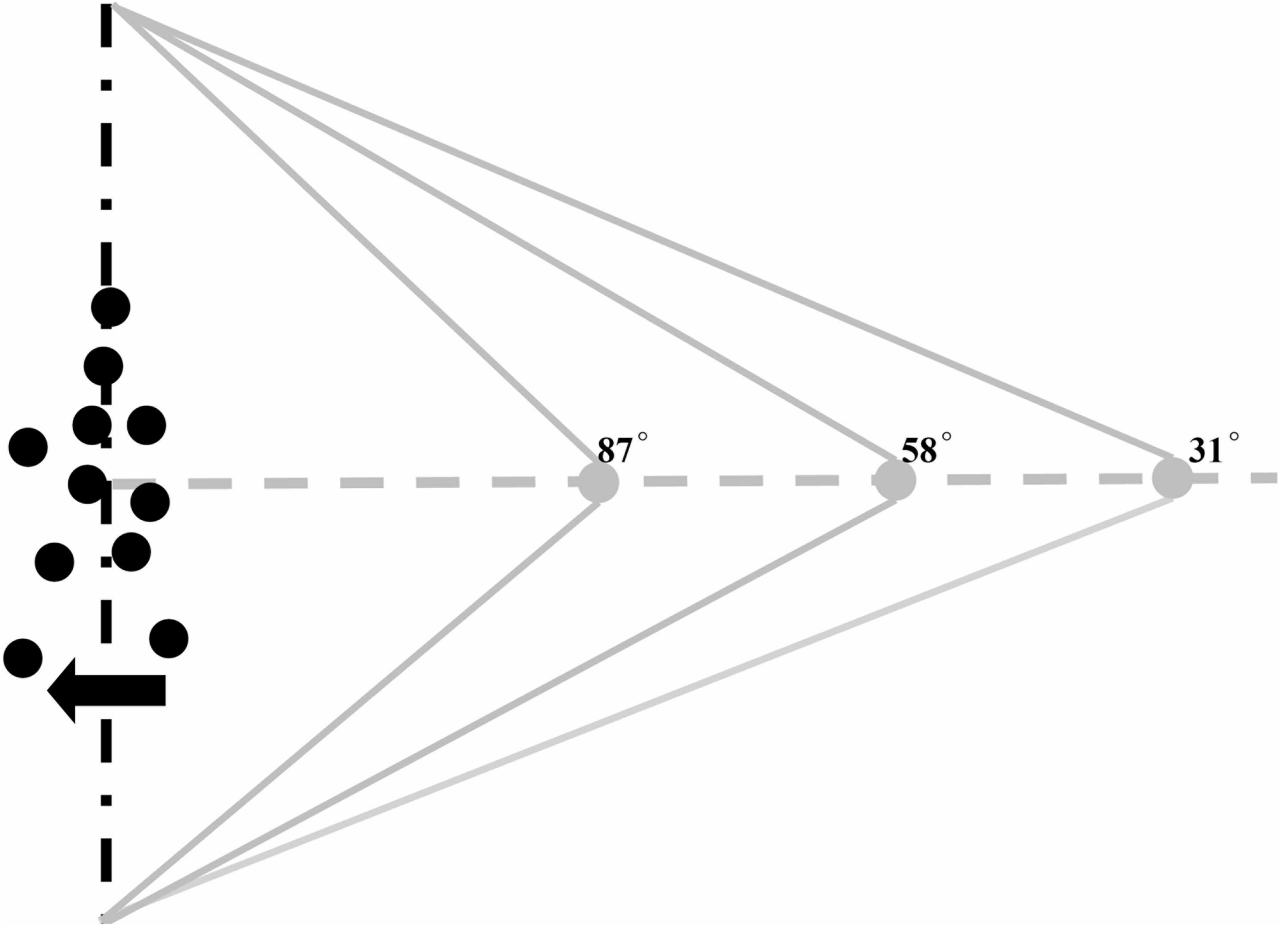
Diagrammatical explanation of the 2-D view of the 3-D projection space. The solid gray lines represent the projection lines of the perspective projections (3 field-of-view angles: 87°, 58°, and 31°). These three angles were determined via a pilot experiment (*n* = 8): 58° was the point of subjective ambiguity when perceiving ambiguous BM (i.e., 50% perception of motion towards participants and 50% perception of motion away); at 31°, participants were inclined to judge the BM display as walking towards them, while at 87°, participants were inclined to judge the BM display as walking away. The gray dots represent projection plane. The black dots represent the point-light walker which is walking away from the projection plane.

To examine whether guilt affects perception of approach, we adopted the paradigm of De Hooge et al. [3] to induce guilt in participants (see methods), which has been well accepted in eliciting guilt [3,6,14,15]. Finally, to measure perception of approach/avoidance, we took advantage of a facing-the-viewer [16] (FTV) bias when perceiving ambiguous biological motion (BM). Participants were presented with a rear view of human walkers in a point-light display format, which depicts human movement via a set of light points fixed at designated joints of the human body[17] (see Fig 1 in Experiment 1 for an example). Participants were asked to judge whether the BM visualization was walking towards or away from them. Although the BM display supports both percepts with equal probability, naive participants tend to perceive the BM as facing towards them (i.e., FTV) more often than facing away [16,18–20]. One promising explanation claims that FTV bias occurs for sociobiological reasons: Misperceiving a person as receding when they are actually approaching has more severe consequences than the reverse situation [21,22]. Here we manipulated the identity (victim vs. neutral) of the figure in a display of ambiguous BM. We predicted a significant increase in FTV bias for guilty people relative to neutral-state people; however, we also predicted that increased FTV bias was specific to the BM of the victim.

## Experiment 1: Guilt increases FTV bias for BM of the victim

We first examined whether there was a significant increase in FTV bias for guilty people relative to neutral-state people, when observing the BM of the victim. Participants first completed a color-discrimination task with a partner to induce a particular emotional state (guilty vs. neutral); they then completed an orientation-discrimination task for a walking BM display.

### Participants

Eighty students (40 males; 18–25 years old) took part in the experiment. Half participants (20 males) joined in the guilty condition, the other half (20 males) joined in the neutral condition. All were Zhejiang University undergraduates, who provided signed informed consent and had normal or corrected-to-normal visual acuity, and no mental disorders. They received at least 30 RMB, and 45 RMB at most (through lottery wins; see procedure below). The study was approved by the Research Ethics Board of Zhejiang University, and was performed in accordance with the approved guidelines.

### Stimuli and apparatus

Stimuli were displayed on a 19-inch CRT monitor with a resolution of 1024×768 pixels at an 85 Hz refresh rate. All participants were seated in a dark room, about 60 cm from the screen. Twenty-six capitalized English letters (1.5° × 1.5° of the visual angle) were used in the color-discrimination task. Each letter had two possible colors: red (255, 0, 0 in RGB value) or green (0, 255, 0).

The point-light BM display consisted of 13 points (0.25° each), and subtended about 7° of the visual angle. The BM walker was presented for 4.5 sec, moving as if walking on a treadmill in the screen center, and in reality was facing away from the participant (cf. Schouten et al [18]). Each BM consisted of three walking cycles, and each cycle consisted of two steps. Moreover, across trials the start position of the animation cycle was randomly selected. Three field-of-view angles (31°, 58°, and 87°) were adopted for the BM displays (Fig 1). A Kinect sensor was used to induce participants’ belief that the BM they observed was motion captured from their partner in the guilt condition.

### Experimental design and procedures

A 2 (emotional state: guilty vs. neutral) × 2 (field-of-view angle: 31°, 58°, and 87°) mixed design was adopted, taking emotional state as a between-subjects factor and field-of-view angle as a within-subjects factor.

The experiment consisted of four parts. In the first part, participants were told that they would complete two tasks: The first task would be performed in conjunction with another recruited person (a confederate, role-played by a female student), and the second task would be conducted separately. Before the first task, participants were told that their BM would first be recorded for later experiment. Both the participant and the corresponding confederate stood in front of a Kinect sensor and were required to perform continuous walking for at least 10 seconds. Critically, the participant witnessed the full recording process for the confederate. This process was used to induce participants’ belief that the BM they saw in the later BM task was collected from the confederate. It is of note that the Kinect sensor actually did not record their BM, and no participant doubted the authenticity of this process. After BM recording, the experimenter told the participant that he had to pre-process the collected BM data for a while and suggested that during this period they might have a chat, because afterwards they would participate in a task together. Then the experimenter left the recording room for about 3–5 minutes, and the confederate actively initiated the chat to form a good relationship with the participant.

In the second part of the experiment, participants were induced to have a guilty or neutral feeling towards their partner. The experimenter returned to the recording room and started the experiment. The participant and the confederate were led to two different rooms, and the participant was told that he/she would take part in an online task cooperatively with the confederate. To enhance participants’ feeling that they were involved in a dual-agent task, participants were shown a gray icon in the center of the screen to represent the confederate, and were instructed to wait for the confederate to appear online. After 2.4 to 7.2 s, the gray icon turned into a colored one to indicate the online of the confederate was and the experiment could start. However, in reality, the confederate played no further role in the experiment; the participant completed the task alone. The task was to discriminate the color of the displayed letters (cf. De Hooge et al. [3]). The full task consisted of two rounds: participants were to press the spacebar to red letters in round 1 and to green letters in round 2. Critically, participants were told that while they responded to one color, the confederate responded to the other. In each round, a total of 312 letters in either red (50%) or green (50%) appeared randomly and rapidly (400 ms or until response) on the screen, with a short (300–400 ms) blank interval between trials. Participants had to respond before the letter disappeared. Additionally, they were informed that they would win a bonus (8 lottery tickets) if their task performance was above 100 hits, and the tickets would offer them the chance (in a lottery) to win extra money (0–15 RMB). Furthermore, to prevent participants from responding to both colors, they were told that their total scores were calculated by the number of correct hits minus the number of false alarms. At the end of each round, total scores were shown on the screen. It is of note that the scores were pre-defined regardless of participants’ actual performance. Feedback was provided on hits. However, since the task was somewhat difficult due to rapid presentation of the stimuli and thus the displayed score was usually higher than the participant’s actual score, to ensure participants did not doubt the authenticity of their score, we added a certain number of instances of feedback for incorrect trials, such that the overall number of instances of correct feedback was close to the displayed score. After finishing the first round, participants received bogus feedback that they had earned the bonus as a result of both their own good performance and their partner’s. Critically, in the second round the bonus for each person was determined by their partner’s performance. In the guilt condition, the participant was told that the confederate had not received the bonus because of the participant’s poor performance, while the participant was awarded the bonus because of their partner’s good performance. In the control condition, the participant was told that both the participant and the confederate had won the bonus. There was a 5-minute break between rounds 1 and 2. This part lasted about 20 minutes.

Once participants had completed the second part of the experiment, they proceeded to the third part: an orientation-discrimination task for walking BM. The participants watched the BM stimuli for at most 4.5 s and judged the orientation of the BM, by pressing the up-arrow on a keyboard for a figure walking away and the down-arrow for a figure walking towards them. Critically, participants were told that the observed BM was based on the data collected from their partner in the first part of the experiment. Thirty trials were presented for each of the three field-of-view angles (31°, 58°, and 87°; see Fig 1), resulting in a total of 90 randomly displayed trials. A short break was given every 30 trials; the whole task lasted about 10 minutes.

Finally, to check the validity of the emotional state manipulation, in the final part participants were required to answer six questions displayed on the screen, five of which related to fundamental elements of guilt [3,23]. These questions were (in Chinese): (1) how responsible you felt, (2) how much you felt that what you had done was wrong, (3) how much you thought about what you had done to your partner, (4) how much you wanted to share your lottery tickets with your partner, (5) how many you would like to share, and (6) what the aim of the experiment was. Participants indicated their answers on a scale ranging from 0 (not at all) to 10 (very strongly), except for question 6. Finally, participants were thanked and debriefed. This part lasted about 5 minutes.

### Results & discussion

**Fig 2:**
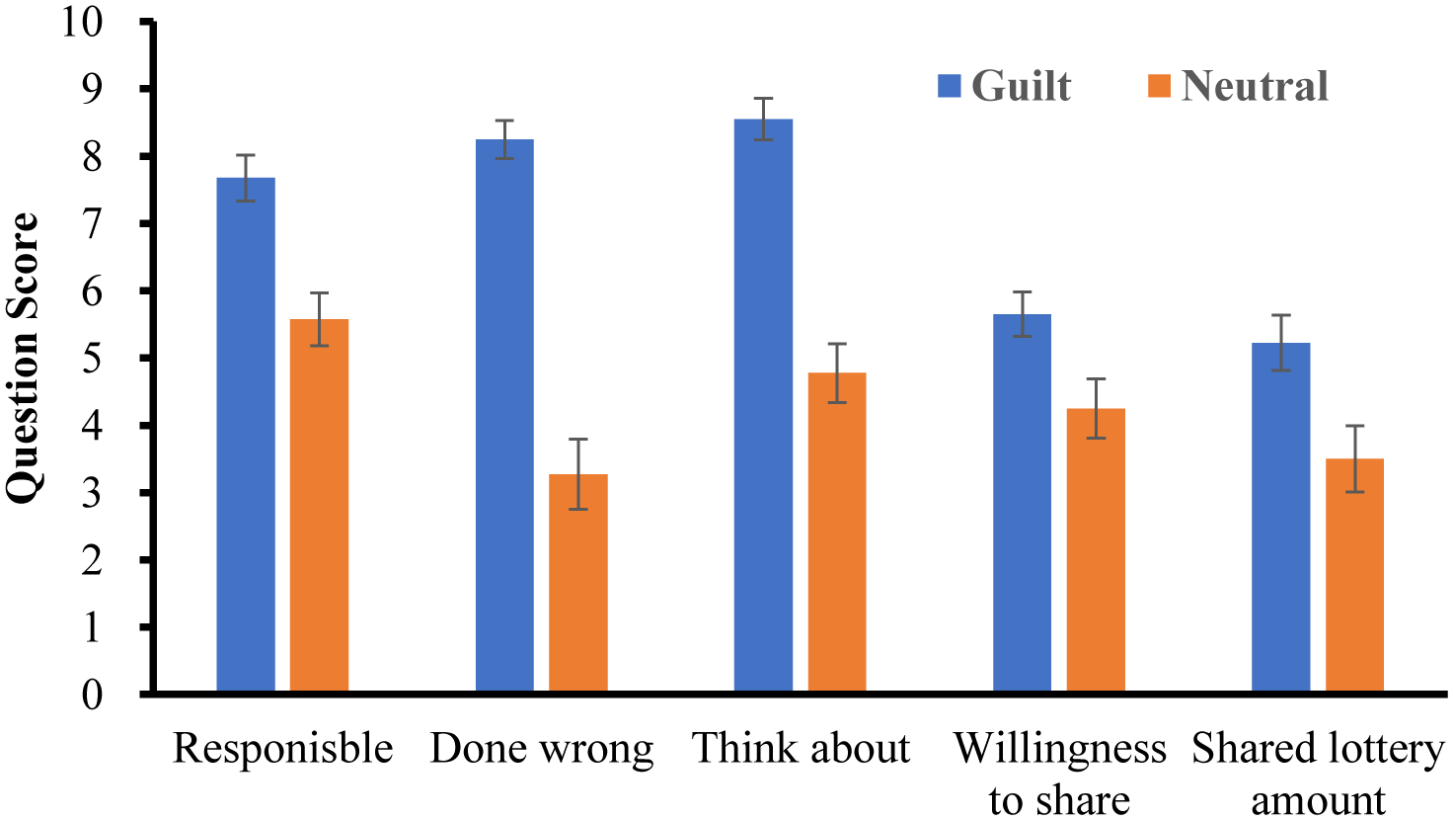
Score for the first five questions in Experiment 1 (error bars represent standard errors).

We first examined whether the emotional state manipulation was effective. A significant main effect of emotional state was found in all of the first five questions (see Fig 2), all *p*s *<* .05, *Cohen’s d* > 0.55, suggesting that the manipulation of guilt was effective. Additionally, no one correctly guessed the purpose of the experiment.

**Fig 3:**
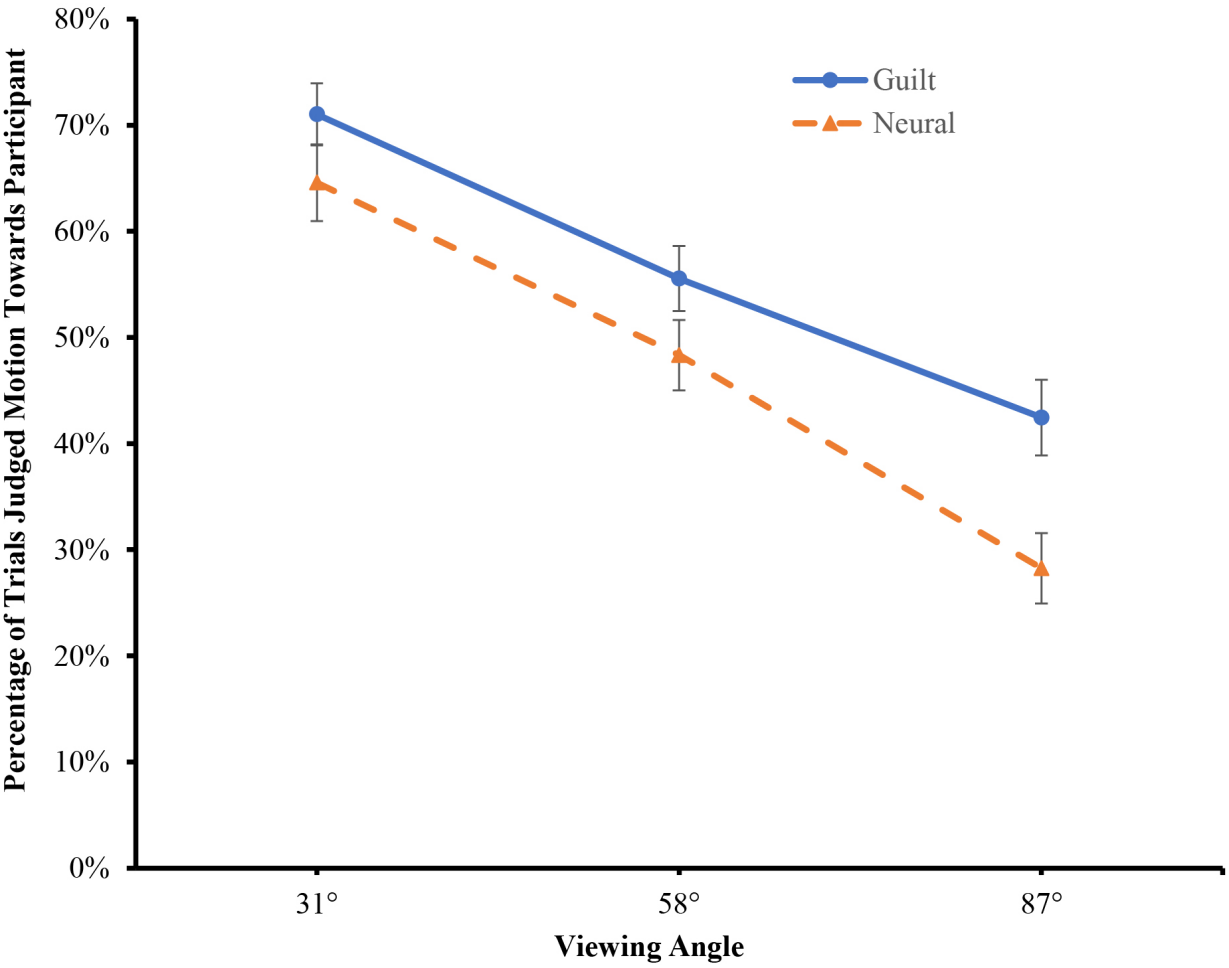
Results of Experiment 1 (error bars represent standard errors).

We then conducted a mixed two-way analysis of variance (ANOVA) on the percentage of BM visualizations judged to be walking towards the participant (see Fig 3). The ANOVA revealed a significant main effect of field-of-view angle, *F*(2,156) = 88.062, *p* < .001, *η*^*2*^_p_ = .530. Post-hoc analysis (Bonferroni-corrected) revealed that the degree of walking-towards perception increased from 87° to 31° (*p*s < .01). Critically, the main effect of emotional state reached significance, *F*(1,78) = 6.332, *p* < .05, *η*^*2*^_p_ = 0.075, and this effect was not modulated by field-of-view angle, *F*(2,156) = 1.225, *p* = .297, *η*^*2*^_p_ = .015. Thus, participants exhibited a larger FTV bias in a guilty state than in a neutral state, supporting our hypothesis that guilt affects perception of approach/avoidance.

Finally, we analyzed the correlation between check question scores and percentage of trials judged motion towards participants. To achieve this goal, we first transformed the guilt scores under each question into *Z* scores and then adding all the five scores together for each subject. We found that the Pearson correlation between the two factors was not significant, *r = 0*.*004*, *p* = .*979*. While this finding may be true, we cannot refute the alternative that the current measurement of guilty feeling is not sensitive enough to reflect the real level of guilt feeling, which needs to be verified in future studies.

## Experiment 2: The influence of guilt is constrained to the victim

Experiment 2 further examined whether the influence of guilt over perception of approach/avoidance was limited to the victim. We re-ran Experiment 1; however, we did not tell participants that the BM data were collected from their partners during the orientation-discrimination task. If the influence of guilt was limited to the victim, then no guilt-modulation would be observed.

### Participants

Fifty-four new students (25 males; 18–25 years old) took part in Experiment 2. Half participants (13 males) joined in the guilty condition, the other half (12 males) joined in the neutral condition. When conducting the orientation-discrimination task in the third part of the experiment, we did not inform participants that the BM data had been obtained from their partners. All other ones were the same as in Experiment 1. The study was approved by the Research Ethics Board of Zhejiang University, and was performed in accordance with the approved guidelines.

### Results & discussion

**Fig 4:**
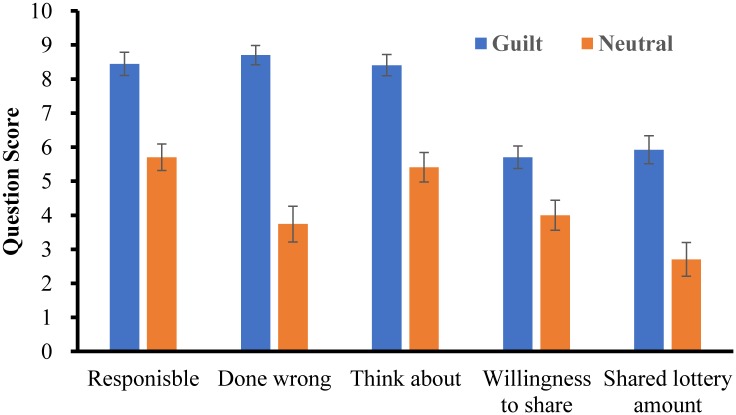
Score of each question in Experiment 2 (error bar stands for standard error).

In line with Experiment 1, a significant main effect of emotion-state was revealed in all the first five questions (Fig 4), all *p*s *<* .01, *Cohen’s d* > 0.70, suggesting that the manipulation of guilt effective. Similarly, no one correctly guessed the purpose of the experiment.

**Fig 5:**
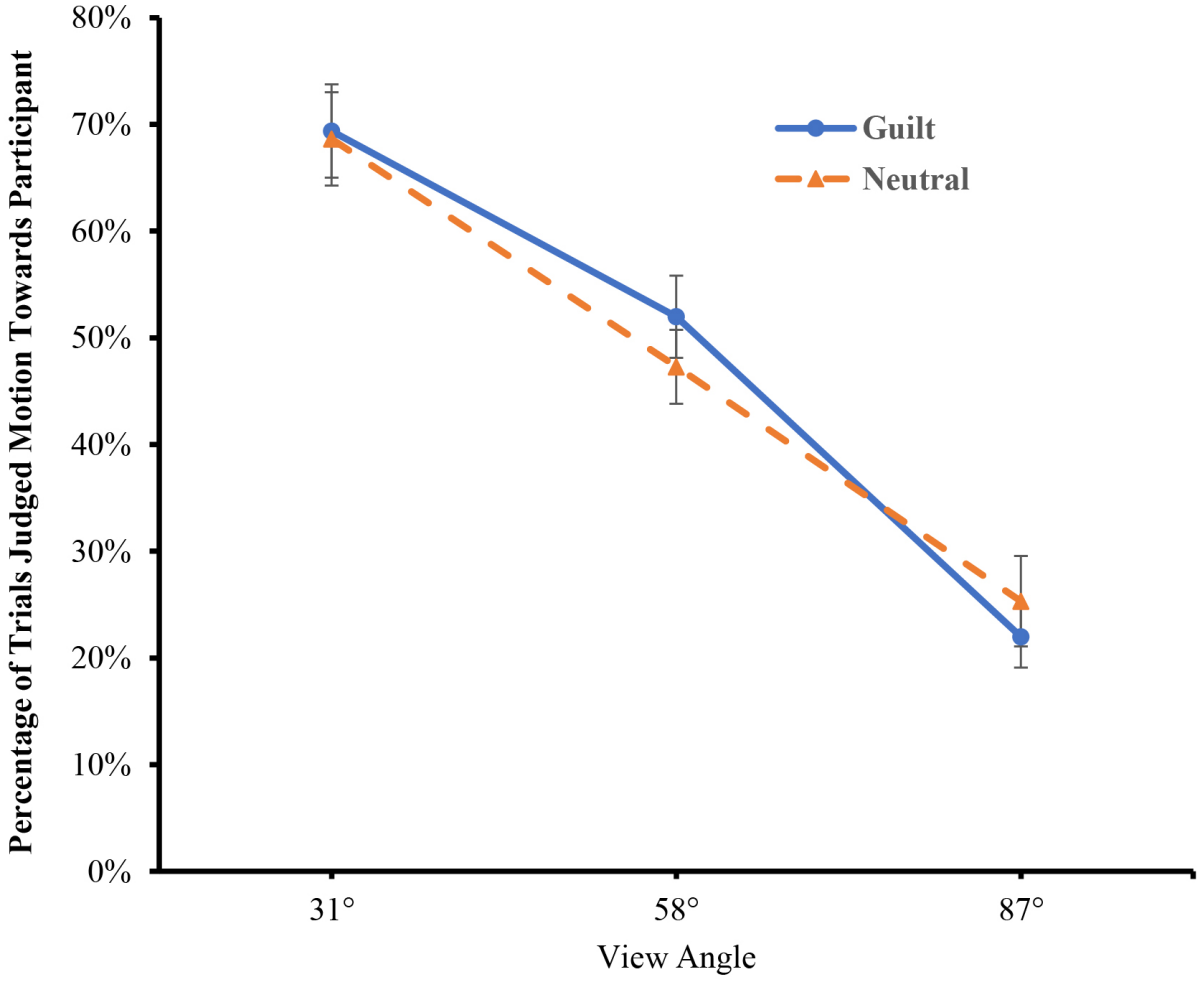
Results of Experiment 2 (error bars represent standard errors).

For the FTV bias, the ANOVA revealed a significant main effect of field-of-view angle (Fig 5), *F*(2,104) = 95.387, *p* < .001, *η*^*2*^_p_ = .647. Post-hoc analysis revealed that the degree of FTV bias increased from 87° to 31° (*p*s < .01). However, neither the main effect of emotional state, *F*(1,52) = .028, *p* = .868, *η*^*2*^_p_ = .001, nor the emotional state × field-of-view angle interaction, *F*(2,104) = .741, *p* = .479, *η*^*2*^_p_ = .014, was significant, suggesting that emotional state did not affect FTV bias.

## General discussion

This study is the first to investigate whether guilt affects visual perception, hypothesizing that guilt would influence perception of approaching the guilt-related victim. We tested this hypothesis by inducing different emotional states via a color-discrimination task, and then measuring their influence on perception of approach/avoidance indexed by an FTV bias. In line with the prediction, we found that guilty participants were more inclined to judge the ambiguous BM to be walking towards them relative to neutral participants (Experiment 1); however, this modulation was limited to perception of the victim’s BM (Experiment 2).

The present study investigated the influence of guilt from the perspective of approach/avoidance perception. Previous studies predominantly focused on the influence of guilt on high-level behaviors [1,2], and only one recent study attempted to explore the relationship between guilt and cognitive processing [10]. The present study is the second study directly exploring the influence of guilt on cognitive processing, adding new evidence for the view that guilt not only affects our external behavior, but also considerably impacts our internal cognition. Moreover, in contrast with Cavalera and Pepe [10], who investigated working memory, the present study for the first time documents the influence of guilt on lower-level perception.

This study further demonstrates the necessity of exploring the relationship between moral emotion and different levels of cognitive processing. Indeed, extensive studies have been conducted on the relationship between non-moral emotion (e.g., anger) and cognition, and have found that non-moral emotion has a considerable influence on distinct levels of cognitive processing [24,25].

According to the multi-component model of emotion [13], guilty individuals generate redemptive motives, leading to prosocial behavior. Therefore, guilty people may tend to compensate their victims for the resultant harm. Meanwhile, it has been revealed that guilt is linked with an increase in self-esteem [26], improvement in communicative skills [27] and tendency to empathy [28], all of which may promote tendency to approach the victim. Brain-imaging studies have also shown that guilt activates the left prefrontal cortex [29], which has an intimate relationship with approach/avoidance motivation [30]. However, no study has directly examined whether guilt indeed affects tendency to approach. The current study closes this gap by offering clear-cut evidence supporting this link. Our finding implies that the motive for compensation affects perception of approaching motion.

Furthermore, in line with De Hooge et al. [3], the current study adds new evidence supporting the view that the influence of guilt is specific to the victims. Both Experiments 1 and 2 effectively induced feelings of guilt (no difference was found between the two experiments on guilt; all *p*s > .05 in the first five questions); however, the increased perception of motion towards the participant was only manifested in Experiment 1, in which participants were informed that the BM was that of the victim although in reality the BM had no relation to the victim at all. Moreover, no participants had correct assumptions about the purpose of the experiments, implying that our results were not influenced by high-level cognition that relates to task demands [31]. Therefore, the present study implies that guilty participants have a strong willingness to compensate their victims, and suggests that guilt exerts a top-down influence on lower-level perception. It is worth noting that the current study used a BM from a third neutral person, and the participants were told that the BM was from the victim considering that the participants were not familiar with the victim. The results of Experiments 1 and 2 together suggest that this setting of BM was successful. However, if the participants were very familiar with the victim, the current manipulation may not work. This is because each individual’s BM carries a dynamic motion signature that tells the identity, and human beings are good at extracting the identity information from BM [32]. Consequently, under such a circumstance of familiar relationship, we predict that the guilt works only when the BM is from real victim. Finally, in contrast with guilt driving people to focus on the victim, those in a state of shame usually focus on themselves [1]. Future study may consider elucidating the influence of shame on approach/avoidance perception in future studies.

## Supporting information

S1 Dataset All data underlying the findings reported in article. (RAR)
